# Environmental and Moral Education for Effective Environmentalism: An Ideological and Philosophical Approach

**DOI:** 10.3390/ijerph192315549

**Published:** 2022-11-23

**Authors:** Abida Begum, Jingwei Liu, Hina Qayum, Amr Mamdouh

**Affiliations:** 1 College of Marxism, Shanghai Lixin University of Accounting and Finance, Shanghai 201215, China; 2 Heilongjiang Province Think Tank for Ecological Civilization Construction and Green Development, Harbin 150040, China; 3 Elementary and Secondary Education Department, Peshawar 25000, Pakistan; 4 Architectural Engineering, Faculty of Engineering and Technology, Future University in Egypt, New Cairo 11835, Egypt

**Keywords:** environmental education, moral education, environmentalism

## Abstract

This article offers a critical review of the research on moral and environmental education as a basis for building environmentalism. The review’s objective is to present an ideological and philosophical theory and research on environmentalism through moral education. The bulk of this research involves empirical research that examines the correlation between moral education and environmentalism, ideologies produced by moral education, and philosophical arguments inherent in environmental education. A deductive argument is made following the review of the existing research on moral education to highlight the educational approaches that have been hailed as effective. Some of the environmental educational approaches identified as being effective include proactive environmental education, creating an environmentally conscious environment, and real-life environmental education simulations. The research also identifies moral education whose focus is the creation of a moral consciousness among learners as being critical for the development of environmental consciousness. Inculcation of religious education, creating a moral educational atmosphere, moral leadership, moral life simulations, and use of Ubuntu and Ukamu theoretical frameworks will bolster building a moral consciousness among learners. The argument presented in this article is that despite the existence of some contrary research, moral education can act as a bolster to positive attitudes, actions, and behaviors towards the environment.

## 1. Introduction

Effective management of the environment is a product of a deep understanding of the environment and the interdependence of the various factions that are determinants of environmental health. The world’s existence and its continuity are crafted in a delicate balance that is made of interdependent elements. The interdependent life and non-life forms can be classified into ecosystems and habitat constituents that sustain man, plants, and animals [1]. Environmental issues constitute a disruption of this balance. Climate change has been singled out as one of the most problematic issues for the global community [2]. This problem is rooted in the poor management of the environment and its resources, leading to negative consequences for people, animals, and plants now and in posterity. The environmental issue is cosmopolitan, incorporating different aspects where changes omit the living and nonliving matter. Some of the critical environmental issues include water availability, biodiversity, forest cover, carbon emissions, extraction of natural resources, food security, pollution of water and air, rainfall, and desertification, etc. [2]. Further, a lack of prudence in natural resource utility is destroying the earth through actions such as extraction, trade, and the burning of fossil fuels such as coal and oil. Global consumption has also been increasing, and this has caused a strain on natural resources [3]. The issues affecting the environment have their genesis in modernity, which is a complex nexus of political, legal, societal, capitalistic, and cultural positions [4]. This makes it difficult to have a coherent approach to environmental education owing to conflicting interests.

The current environmental issues identified pose an urgency to the global community. However, the actions being taken to mitigate the environmental issues are insufficient in comparison with the existing global environmental challenge. The problem lies in the gap that exists between measures being undertaken to maintain environmental sustainability and the devastation that the world’s environmental degradation is causing. There is a knowledge gap among global citizens regarding the actions that they should be taking, as they are often unaware of the individual actions that contribute to global environmental sustainability. A radical change is needed in education so that an individual can develop a moral consciousness that remains steadfast. According to Albulescu [5], subjective thinking and personal choices are influenced by the conscience.

### 1.1. Values and Reflection in Environmental and Moral Education

Developing an environmentally conscious society requires educating society on the importance of the environment and getting people to act ethically. Gamayunova and Vatin [6] stated that environmental education is meant to highlight that morality is not only domiciled within social relations but also encompasses our responsibility towards future generations, animals, and other forms of life. Institutions of learning are one of the most critical and ideal areas where environmental education and environmental ethics can be taught. They form a microenvironment and act as cradles for the enlightenment and development of future leaders. They need to be on the frontline in educating and implementing environmental sustainability.

Lapuzina et al. [7], noted that in institutions of higher education, the level of awareness and commitment to environmental ethics is low, with most students viewing it as an abstract social value. In research that was conducted at University Alpha, Castro and Jabbour [8] found that there was only a partial implementation of the Sustainability Campus Framework. A similar study was conducted by Dagiliūtė and Liobikienė [9] at Vytautas Magnus University, Lithuania, and it was found that the university was not environmentally committed to sustainability. The university’s policies on the environment were pedestrian, and its practices were inconsistent. There is also a gap in environmental education among young children in grade school. In an investigation on the understanding and the views of children on the environment, Littledykes [10] found that most children have limited knowledge and understanding. Littledykes [10], Dagiliute and Liobikiene [9], and Castro and Jabbour [8] all pointed to the discrepancies that exist in the education system from the formative stages to the university level about environmental sustainability. Some people possess the knowledge of the actions that should be taken toward sustainability, but they lack the moral consciousness to drive them toward these actions. Ivan [11] explained that moral consciousness constitutes a rule of morality, which acts as a guide in decision making. According to Geiger et al. [12], the absence of moral consciousness and behavior has led to the degradation of the environment. Jie [13] said that deficient knowledge, low commitment, and lukewarm perception towards the environment are inherent in the inefficiency of environmental and moral education partly due to the inherent passivity of moral education, which makes morals appear remote, general, and vague. This points to the glaring need for a more efficient environmental and moral education given that grade schools and institutions of higher learning are important for the acquisition of critical values and behaviors toward sustainability.

Moral education can equip individuals with intellectual principles. There is a need to create a moral environment within which moral consciousness can develop. Moral education should be accompanied by moral leadership. Institutional administrators and instructors in all subject areas should be beacons of morality to inspire the learners to emulate them [14]. Ineffective moral education is to blame for the poor moral grounding in society [13]. Effective moral education should be an agent of social change, and this should be reflected in individuals’ acquisition of moral consciousness. El-Hani and Mortimer [15] defined moral consciousness as a combination of cognitive structures and intellectual principles that helps in the deduction of what is right and what is wrong. This consciousness is driven by self-motivation and helps to redefine the relations with the self and other people. An individual needs to internalize certain standards about right and wrong to make autonomous moral decisions. An individual with a moral conscience will have a self-imposed and self-assumed mental framework for decision making after the acquired moral conscience precipitates into a psychological conscience [9]. Moral consciousness also guides personal actions in a way that they are attuned to the values, principles, and norms of a society. One of the most effective moral education techniques is the values clarification technique [13]. This moral educational technique is instrumental in eliminating inner conflicts about moral issues through the use of persona analysis. The major techniques of value clarification technique include value grid, value focus, rank order, value survey, and either-or-focused choice [16]. Through the application of these techniques, learners’ attitudes and intrinsic motivations are changed, and they become more aligned with moralistic values.

### 1.2. The Rationale of the Study-Environmentalism

The remedy to the environmental problems identified lies in environmentalism. Environmentalism is a conglomeration of actions and set of attitudes that are meant to safeguard the environment, restore the damaged environment, and improve the environment. Of significant interest in environmentalism are water resources, quality of air, and protecting ecosystems as well as natural habitats that are home to humans, plants, and animals. The global society exists on a planet with finite natural resources, and human existence intertwines with the sustainability of finite natural resources. This makes environmentalism critical for the long-term sustainability of the planet. Environmentalism is executed through political, economic, and social activism, which has the objective of safeguarding the environment from irresponsible utility and conduct. Such activism leads to mitigating factors that include policies and legislation, the consumption habits of individuals, and the lifestyle of people.

The basis of environmentalism is that nature has been personified and is perceived as a singular entity. Cano Pecharroman [17] argued that an entity can possess legal rights even if it cannot exercise its own will or have any interest. He further argues that these rights are not reducible in the face of opposing rights of other entities. Jasanoff [18] said that all entities that have legal rights should be recognized in the legal system, and as such, any damage done to them should be subject to consideration through the due process of the legal system. If the entities are not able to defend themselves, they should be defended by legal institutions and other watchdogs. A balance needs to be established between the organizations that will safeguard the interests of vulnerable entities and the needs of citizens. This is to avoid the overbearing burden that environmentalists may impose on society while ignoring the welfare of society. Conversely, the exploitation of the environment is a profitable venture, and some political factions work to undermine environmental regulations so that they can exploit natural resources.

Environmentalism underscores the necessity for the protection of the earth’s resources and prudence in the utility of the resources that are needed for our survival on the planet. There is a need for the preservation of natural elements and resources since the survival and continuity of humanity are tied to them [19]. Environmentalism is advanced by environmentalists, who are agents of change in their pursuit to protect and restore ecosystems and habitats. Environmentalism is emerging as a profession, where environmentalists act as advocates for the environment at a certain civic or institutional level. The environmentalists help the entities to reach their set-out goals without compromising on the environment or devising ways in which they can advance the environmental goals within their context. Environmentalism faces the conflict that emanates from professional responsibility and moral responsibility. According to Campbell [20], the moral content of every profession varies, with some professions having very low moral content. He further says that every professional has to sometimes weigh what should take preeminence: professional responsibility or moral responsibility. If a professional thinks that the moral obligation outweighs the professional responsibility, then the individual may have to violate the professional responsibility to safeguard certain moral responsibilities. Sometimes, environmentalists have to deal with immoral actions against their very own institutions that have given them their profession to safeguard environmental interests. In such a case, the environmentalists are choosing to override their professional responsibility with their moral responsibility to the environment, for which they are advocates. Environmentalists and other professions have to constantly deal with the dilemma that pits professional responsibility and moral responsibility against each other.

## 2. Aim of the Study

Research indicates that environmental and ethical education has the potential of developing environmentalism among people. Heeren et al. [21] said that when individuals have the right knowledge about sustainability, and this knowledge is coupled with the right attitudes, then it is likely to be translated into positive behavior towards the environment. This study aims to highlight the dominant ideologies that research states are produced by moral education and environmental education. Further, this research examines the existing causality between environmentalism and moral consciousness as presented in different research. The objective is to offer a critical appraisal of the two arguments as possible pathways to developing environmentalism in society and give recommendations on the approaches that should be taken in moral and environmental education.

## 3. Methodology

### 3.1. Identification Phase

We followed established procedures for systematic reviews [22,23] of scholarly literature on the effectiveness of environmental and moral education for better environmentalism. Systematic reviews can incorporate findings from various study designs and aid in navigating research-implementation spaces by encouraging thought, evaluating a variety of evidence, and respecting a diversity of approaches and epistemologies [24]. An extensive search was performed using multidisciplinary databases, including *Google Scholar*, *Scopus*, *Science Direct*, and *Web of Science*. The articles were found using the keywords environmental education, ecological education, moral education or ethical education in conjunction with climate change, global warming, biodiversity, species diversity, species loss, environmentalism, pro-environmental behavior, environmental friendly behavior, ecological behavior, green behavior, green purchasing, and green activities.

### 3.2. Screening Phase

In this phase, we studied each paper downloaded in the previous step and decided whether the paper should be selected or not for inclusion in our study. In summary, scholarly publications were extracted using the search criteria indicated above. After studying titles and abstracts, irrelevant research was eliminated, leaving a total of 77 important studies.

### 3.3. Eligibility Phase

At the eligibility stage, each study was evaluated in depth. The technique extracts the influence potential and the details of each journal’s experiments, which either performed or confirmed those guidelines. Numerous data analysis techniques were implemented to maintain an orderly count during the classification and sorting phase. Online resources and EndNote software were used to store the record collected from the reviewed papers, analyze the data, and make annotations.

### 3.4. Inclusion Phase

At this stage, the researchers performed a descriptive overview of the results extracted to effectively categorize the development plans and organize the encrypted data to answer concerns that influenced the literature review. This analysis shows that the influence of environmental and moral education on environmentalism is an evolving field of research, demonstrating a consistent rise in the number of publications in past decades. Inclusion and exclusion criteria were used to marginalize relevant and non-related research. All studies on environmental and moral education on environmentalism were included in the sample population. Inclusion requirements were based on a focused research topic as a prime precondition. Grey writings, i.e., summaries (included at a conference), compositions, and incomplete articles were excluded from the analysis. The database lists of all the studies were reviewed systematically to conduct such work. The search was not restricted in any way. We also searched the references of previous reviews. Moreover, we did not limit our search to studies published in a certain period. Our initial set consisted of more than a thousand papers. After removing all duplicate, non-peer-reviewed articles, and non-relevant articles, we reached a final set of 77 papers.

### 3.5. Evaluation of the Quality of the Studies

In the last stage of the process, any conflicts between the authors were discussed and resolved. The articles were then summarized, and the process of interpreting and categorizing the papers provided us with a wealth of valuable and stunning information. Given the fact that the methods are indirectly described in the abstract and methodological sections of the articles, the authors had to review the entire content of the papers and look at more knowledge to determine the particular approach used for environmental and moral education impact on environmentalism.

### 3.6. Data Analysis

The studies analyzed the correlation between environmental and moral education on effective environmentalism. All selected papers’ data related to the effect of environmental and moral education on effective environmentalism were evaluated. The results of the previous studies were used to derive the current study results. Analysis was performed based on the relationships among the study variables. We presented a more holistic picture based on critical and supporting arguments, which enabled us to prepare a critical review paper on the effective integration of environmental and moral education as a basis for building environmentalism.

## 4. Results and Discussion

### 4.1. Correlation between Moral Consciousness and Environmental Education with Environmentalism in Research

#### 4.1.1. Correlation between Environmental Education and Environmentalism

Environmental education aims to help build knowledge that will foster environmental sustainability. This capacity is built through the development of cognitive knowledge and a recognition of enlightenment on the place of environmentalism in society. Olli et al. [25] recognized that environmental sustainability can only be attained if people have the necessary knowledge about the environment. Further, ecological knowledge aids in the development of the right attitude and values toward sustainability efforts.

Environmental education is related to consumer-conscious behavior. Through the attainment of the necessary knowledge of products and services and their impact on the environment, consumers develop a consumption consciousness [26]. Through environmental awareness, consumers are persuaded to purchase environmentally friendly products, purchase products that are packaged with green materials, and use products that are grown organically. Individuals are also encouraged to use bicycles and public transport to reduce the carbon emissions from private cars. At an individualized level, environmental education inspires a healthy lifestyle. A healthy lifestyle includes maintaining a low ecological footprint, using resources thriftily, recycling and reusing, better soil management, waste management, sanitation, reducing waste, and reducing the exploitation of finite natural resources. Apart from changing consumption trends, environmental education inspires individuals to take actions that are meant to restore the degraded environment. Environmental education helps learners and citizens to try and acquire values that are important for environmental conservation.

Education on the environment facilitates the exploration of the challenges that are being faced by the environment. Further, education helps in developing awareness about environmental issues, and this is the precursor to developing solutions for environmental challenges [27]. Environmental education helps to inspire activism spanning from civic political machinations on the environment to efforts to reduce wastage of resources, reduction in carbon emissions, reduction of climate change effects, greening of the environment, the perseveration of air and water resources, and use of renewable energy. Activism then leads to policymakers being made aware of environmental issues that are of public interest, which consequently leads to legislation that helps to safeguard the environment. Through environmental education, individuals will be able to recognize how environmental issues such as climate change and global warming pose an urgent challenge for the global community. Contemporary environmentalism helps to deal with environmental challenges at the communal level as well as ameliorate the state of the space the community lives in.

#### 4.1.2. Correlation between Environmentalism and Moral Consciousness

Moral consciousness helps individuals acquire a deliberate and purposeful conscious system of determining what is right and what is wrong. Moral and behavioral consciousness regulates different factors that are instrumental in decision making, which include the environment, existing culture, economic status, and intrinsic motivations [28,29]. Developing moral consciousness among people is instrumental in reviewing personal actions such as the consumption of fossil fuels, which is the largest source of environmental degradation and a major contributor to global warming [30,31].

Moral consciousness develops self-drive for following rules and regulations that govern the society of which they are a part. Saleem et al. [32] recognized that moral consciousness also helps to overcome social pressure while in pursuit of certain ends. By overcoming social pressure, individuals are compelled to take responsibility for their actions and utterances. This includes being sensitive to issues such as the environment and sustainability. Furthermore, moral education can change an individual’s perception and consequently lead to moral action in personal and social engagements. The integration of moral and environmental education is capable of producing better environmental attitudes, behavior, and intentions.

The moral component of individuals inspires positive behavior toward the environment, which then develops into environmentalism. According to Poškus [33], behavior is a cosmopolitan aspect that is a constituent of values, attitudes, personal norms, perceived behavioral control, intentionality, and intrinsic motivations. These aspects, values, and points of view can all be acquired through moral and environmental education. Moral education should highlight the relationship of man with nature and the stewardship role that man has [29,34,35]. Consequently, this steward role can drive man towards sustainability.

Poškus [33] investigated the motivation behind sustainable efforts, which include recycling, conserving water and electricity, and use of friendly transportation, among Lithuanian university students. The research found that personal values and norms are a predictor of behavior toward sustainability efforts. This finding is consistent with research by Poortinga et al. [36], which found that social norms influence the behavior of people. Poškus [33] found that values are stable and are not subject to change based on situations. The research from Poškus [33] also found that attitudes are not a good predictor of behavior since they are subject to change based on situations.

The consequentialist ethical theoretical reasoning is also a motivation for sustainability. The consequentialist theory holds that something is right or wrong based on its consequent outcomes. Environmentalism as an eco-social construct is informed by a causal structure that informs our perception and the consequent actions that we are willing to take as remedial to the pending doom [19,37,38,39,40]. Events such as rising sea levels, melting of polar ice, extinction of species, rising global temperatures, and desertification present a justification for a call to action. The moral component of the causation is strong since moral considerations take into account the effect of environmental degradation leading to a moral justification for sustainability efforts.

Moralistic and rationalistic motivations contribute to sustainability. Frey and Stutzer [41] stated that moralistic motivations are intrinsic, while rationalistic motivations such as the economics of conservation are extrinsic. The two types of motivations should be fostered in that they act in a complementary manner to advance environmental sustainability. On the one hand, extrinsic motivations through economic incentives such as tax breaks for green energy and tradable emission rights tend to motivate people and companies towards sustainability. On the other hand, intrinsic motivation such as morals, harmony, and beauty inspires an individual’s environmental sustainability [41]. Both types of motivations are acquired in environmental education, making it the enabler of environmental virtue acquisition.

#### 4.1.3. Correlation between Ecological Civilization and Moral Education

Ecological civilization is the possession of certain values and developmental frameworks that further the existence of natural ecology, tending towards sustainability, the natural world, and averting devastation. Magdoff [42] asserted that an ecological civilization exists in harmony with systems of nature, exhibits diversity, and has a balanced fractal organization, life cycles, subsidiary, and symbiotic relationships. According to Chen and Zhao [43], ecological civilization exists within governmental frameworks and eco-socialism while contending with transcending capitalism [44].

Blinc et al. [45] stated that it is the moral duty of man to leave the world the same as they found it or to make it better for future generations while noting that man’s moral obligation is to meet their personal needs without compromising the ability of the future generations to meet their needs as well. The international community recognizes this mandate. Their effort is reflected in The United Nations Environmental Program (UNEP), which has 17 goals that seek to address the challenges that range from climate change to rampant poverty, and they are all anchored in creating sustainable development [46]. Their 2030 agenda is to protect the planet from degradation through sustainable management of resources, production processes and consumption, and action to mitigate climate change.

The level of ecological awareness, respect for nature, restoration, and protection of the environment needs to be developed across the world [47]. The pathway toward ecological civilization will be charted through the creation of a moralistic society that understands what needs to be done to create a sustainable future. Moral education and ecological responsibility should start as soon as children are enrolled in grade school. This early instruction and molding will ensure that children develop from level 1 where they lack norms, grow through level 2 where norms are developed by their instructors, and finally become part of level 3 where norms are internalized [48]. Ecological civilization needs to be developed into an ideological education, which should be integrated into the courses learned in universities and colleges [49]. The university stage is a value-forming life stage and a personality-formation stage as well. The focus should be to enable the learners to develop values that will inspire them towards ecological contribution and activate their sense of responsibility and sustainable living. Chang, et al. [50] said that instructors should inculcate environmental education into the social life of the learners to stimulate ecological consciousness and direct their ecological conduct. Zhang [47] noted that in an increasingly digitized and technologically advanced world, there is a need to integrate technology into environmental learning to capture the essence and accrued benefits of technology in environmental education and applications.

### 4.2. The Importance of Moral and Environmental Education for Effective Environmentalism

#### 4.2.1. The Necessity of Environmental Education in Nature

Environmental education and planning are important to safeguard the future of mankind on the planet. According to Callicott [49], the integrity and the stability of human civilization are at stake. Most peculiar among Callicott’s concerns is that the world is heading to another mass extinction event. Callicott [49] stated that mankind is not likely to survive until the end of the first century of the 3000 millennia. This prediction is based on explosive population growth, the scarcity of resources, runaway global warming, and climate change. There is a need to reevaluate the place of wo/man in his daily existence and the implications of their actions on the grand existential system [51]. Attention should be given to a shared future, where the community and individuals are seen as entangled with nature in a reciprocal partnership. Significantly, Bonnett [51] argued that attention should be drawn not just to the human world but also to non-anthropocentrism as a remedy to the threats to nature. Human discussions need to be accompanied by discussions of the place they exist in, as they are intertwined, eliminating the othering of nature.

The attitude towards labor and environmental protection is bolstered by environmental education, leading learners to develop a desire to take care of the environment, participate voluntarily, and defend environmental actions autonomously [52]. Environmental education based on natural experiences, such as being in forests and bushes, help to develop non-anthropocentric perspectives [51]. Further, the experiences enlighten learners on the interconnectedness of nature and man’s existence while highlighting the flaws in anthropocentric thinking. Learners also acquire a goal-oriented and planned mental framework in their approach to the environment, where they get a chance to develop eccentric models [52].

Environmental education inspires environmentalism within society. Eichinger et al. [53] found that sustainability efforts through education lead to greater efficacy in energy use, effective use of materials, reduction in several utility costs, reduction in the carbon footprint of the campus community, reduction in the amount of waste produced within the campus, increased awareness among the campus community on sustainability, and the creation of a greener campus and image. There is low awareness of professional values that are congruent with environmental ethics. As such, there is a need for a set of principles that sets the moral boundaries to act as a guide for professional and personal responsibility [48]. Educationists, policymakers, and instructors set environmental ethics boundaries through environmental education. This aids in offsetting a professional’s psychological setting of environmental ethics as an abstract concept to professional competence and personal value [54].

Through education on environmental ethics, it is possible to safeguard environmental entities that are vulnerable including the forests, the oceans, the land, and the climate. The above elements are precursors for agricultural productivity and wealth from the seas, which are critical for human survival. Having adequate food reserves is a large component of sustainability. There is a need for recognition that the threat to nature is also a threat to humanity. Callicott [49] stated that it is only through environmental ethics that the stability, integrity, and future of the globe will be safeguarded.

Environmental education is also a tool for dealing with many world problems. Some of these problems include global warming, desertification, climate change, and the extinction of species. Baker et al. [14] recognized that environmental education changes the behavior of people and helps develop plans to bolster sustainability. Jickling and Spork [35] said that many of the problems that are being faced in the world, especially social and environmental challenges, are due to individuals not asking themselves about the ethical implications and consequences of actions being undertaken. Asking ethical questions can help to spur discussions about sustainability and conservation [55].

#### 4.2.2. The Necessity of Moral Education in the Nature

Taneja and Gupta [56] stated that past environmental efforts have for the most past focused on sensitization on environmental issues and that there is a need to inculcate the ethical component of environmentalism to spur actions that safeguard the environment. The purpose of moral education is to craft moral behavior and moral consciousness among people since values are produced by moral education [57]. This association is based on the tenet that there is a complementary duality between cognitive faculties and behavior. Moral education is a means towards presetting the cognitive, leading to an acquisition of predispositions of what is right and wrong.

Moral education highlights one of the significant moral issues, which is environmental injustice. Many of the poor depend on environmental aspects such as rainfall and forests for subsistence. Degradation of the environment is therefore injustice and leads to a proliferation of inequality. Major industrial nations such as the USA, China, and Germany have been emitting most of the greenhouse gases. However, the larger cost of this damage is on the developing countries, as they have to try to balance economic development with environmental needs. Environmental ethics turns to industrialized nations for admonitions. The industrialized world must pay a greater share of responsibility for mitigating climate change and global warming trends [58].

Moral education is a lighthouse in creating a secure global environment and directing actions meant to develop the economy, society, and the environment. Moral education teaches interdependence, cooperation towards the attainment of certain shared goals, and living in harmony with other people and the environment in which they live. Education and moral consciousness are instrumental in the formulation of strategies to reverse the effects of runaway climate change and global warming, which is threatening food security, ecological balance, and natural habitats. Moral education also extends to trade, as identified by Ha-Brookshire et al. [59], who said that it helps to train current and future professionals so that they can build sustainable companies and supply chains that cut across the globe. Begum et al. [31] noted that pro-environmental behavior is developed as a result of moral education. Further, moral education leads to a recognition of the common future of mankind, which leads to a global partnership [60]. It is a time of ecological devastation, as identified by Narvaez [61], and the only salvation for mankind is raising a generation of people who are virtuous and connected to their world. Moralistic individuals will reverse the modern problems that have been caused by self-centeredness, aggression, alienation of man from his environment, and disconnect from the natural world. Moralistic individuals will exist in concert with the ecologies, advancing sustainability across the globe [61].

### 4.3. Counter Argument

Some researchers have claimed that environmental education does not translate into actions that are focused on sustainability. Heeren et al. [21] performed research in the USA among university students, and they concluded that knowledge has a weak correlation with behavior. According to him, knowledge is a weak predictor of the behavior of people. However, he did discount the presence of a combination of knowledge with social and psychological factors as an influence on behavior. Similar research was conducted by Hasiloglu and Kunduraci [62] to determine the correlation between environmental awareness and consequent behavior and practices among learners. The research found that even if learners had high scores on the Attitudes Toward the Environment Scale (ATES), these scores were not reflected in their practices and behaviors towards the environment. The absence of positive behaviors and attitudes towards the environment was interpreted as a lack of environmental awareness.

Gifford and Chen [63] investigated the reason behind poor environmental attitudes and behaviors. The research found that there were psychological barriers that prevented the conversion of environmental knowledge into consequent behaviors. Heeren et al. [21] also indicated that there is a weak correlation bivariate between behavior and knowledge according to research they conducted, concluding that knowledge cannot be a predictor of behavior. Shove [64] said that efforts to change individual behaviors are not successful in that they are focused on changing individual beliefs as the precursor to changing behavior. Many young people do understand the importance of environmentally friendly practices. However, many of them see the dangers that are posed to the environment as being the larger aspects that contribute to global pollution and not individual actions, which are local [65]. Some believe that their actions safeguard the environment of the future, according to research by Meinhold and Malkus [66]. Myers et al. [67] asserted that environmental education is an effective tool for the development of knowledge and positive attitudes toward the environment. However, they noted that this knowledge does not translate to behavioral change. The disconnect identified by the researchers did not lead them to proclaim that it is futile to conduct environmental education. This gap can be sealed by the finding of research by Heeren et al. [21]. According to Heeren et al. [21], as much as knowledge is not a predictor of behavior, if it is accompanied by appropriate norms and values, it has the potential of influencing the behavior of individuals. The challenge is identifying an effective approach to environmental education that will result in greater environmental awareness and consequent behavior. Based on the arguments presented and the countering argument, a holistic environmental education not only encompasses environmental knowledge but also advances moral education so that the two facets can complement each other.

## 5. Recommendations in Research for Moral and Environmental Education

### 5.1. Recommendations for Holistic Moral Education

The effectiveness of moral education is its ability to develop a moral consciousness. Moral education should also focus on building a moral consciousness. One of the effective moral education approaches is religious education. Religious education is an instrumental tool for moral education development and consequently leads to the development of moral consciousness. Religious education seeks to shape individual reasoning and feelings to produce cognitive behavior that is moralistic. When an individual has internalized religious morality, it acts as a catalyst for observation of norms through the providence of motivational support. In the scriptures, there is moral consciousness that is manifested in followers of the different sects. This moral conscience is acquired by individuals when they are assimilated into the scriptural teachings and practices that accompany certain religions. In the research conducted by Estrada et al. [68], 55% of the individuals who practiced moral behavior attributed it to their religious affiliations. This finding indicates that moral beliefs found in religious education are the recipe for moral behavior among some people. Religious practice is cyclic, and this makes religious principles dwell on the conscious level, strengthening moral principles.

Creating a moral atmosphere within the school and the community where certain values are being forged can foster the development of moral consciousness [69]. This is because morals are acquired by individuals from their environment and the content of their moral education. According to Araujo and Arantes [37], values are not predispositions in people, and neither can they be easily internalized, but they are rather a product of a continuum of socialization through objective and subjective actions. Through day-to-day association with other people, lived experiences, and internal musings, individuals get to adapt and practice certain values. Further, Araujo and Arantes [37] said that individuals should be understood from the naturalistic approach, which purports that human perception of reality is a derivative of different social contexts. If there is a culture of morality, and ethical principles guide the actions of individuals in a certain environment, then the inclinations of the members of the community will tend toward moral personhood [51]. This position is supported by Aristotle, who said that the actualization of virtues in an individual is a product of the support and impressions made on an individual by the community of which they are a part. One of the ways of helping the community to own up and craft its moral direction is through periodic forums that interrogate their moral direction and craft projects that reinforce their moral direction [37]. These forums should include all members of the community, including the NGOs, the management, staff, and students, among others. Ethical themes that emanate from these forums should be integrated into the learning framework and the community engagements.

Moral education should be accompanied by moral leadership to create a consciousness of morality at an institutional level. Institutional administrators and instructors should be beacons of morality to inspire the learners to emulate them [26]. Bacchini et al. [39] researched the effect of exposure to deviant contexts on moral grounding among teenagers. The research found that the students exposed to high deviance ended up exhibiting moral decadence, the genesis of which is in peer groups. This finding confirms the postulation of the domain theory that states wo/man’s behavior is shaped by the domains of which they are a part [40]. Individual actions are subject to change based on domain and are constantly molded by justifications to escape harm, administer justice, and ensure the welfare of the subjects involved [38]. Walsemann et al. [70] said that it is important to integrate values in the formative years of children and learners since their formative values have a residual effect in later years.

Moral education and reasoning should be extended to all areas of life, including leisure activities. Leisure is a large part of learners’ lives, and it should be used as an avenue for creating a moral consciousness. Kowasch and Lippe [19] claimed that the scarcity of inquiry into the ethics of some leisure practices is partly to blame for the low social and professional preparedness of students. An awareness of the intricacies of leisure activities in the environment should be created to enable students to be prepared to use moral judgment in recreation activities. Further, education should be advanced through a sociological critique of leisure activities. This will aid in the growth of environmental consciousness and reduce the pedestrian reasoning influenced by the diversity of factors.

There is a need to simulate real-life applications in moral education to equip learners with real-life capabilities and develop their moral consciousness. Often, moral reasoning on environmental sustainability is plagued by moral dilemmas [71]. Indecision on what action to take can be eliminated through an approach where knowledge is not mere indoctrination but a proactive process where learners construct meanings and develop critical thinking and problem-solving skills [71]. As such, there is a need for the creation of an ecosystem where holistic thinking can be fostered through knowledge and action-based learning. Požarnik [71] carried out research on moral reasoning in environmental dilemmas among 11–15-year-olds. The context of the research was investigating the decisions that the learners could make if faced with a moral dilemma that involves suitability efforts as well as economic factors of the individuals involved. Polzarnik’s research found that the learners were unable to make decisions when confronted with moral dilemmas. This is a reflection of the gaps in moral education, which does not equip learners with the capability to process real-life situations that learners will encounter despite their extensive moral education.

Moral education should be structured in a way that will facilitate harmony in the development of religious morality and moralistic consciousness. Religious values are a complement to moral education. This is because moral education equips individuals with intellectual principles, while religious morals develop affective states. This makes the two sides work in coherence to bring about unity in actions and thoughts. There is a need for an interdisciplinary approach to moral education, as identified by Denisa [57]. This is because it is not only in the religious and educational sector that morals become impressed on the individual, but it also happens within the cultural predisposition, artistic representation, and sporting field, among others. Denisa [49] recognized that moral education does not teach all the moral grounds that one is supposed to adopt. However, it does create a suitable internal environment that facilitates the internalization of the aspects that constitute social morality. This happens through a systematic change in the personality structure of an individual, which is the driving force of moral conduct [70].

### 5.2. Morals Consciousness Development through the Ubuntu and Ukamu Concepts

Values and moral consciousness are inherent in the internalized value system, such as Ubuntu. The Ubuntu concept of humanness, which has its genesis in Sub-Saharan Africa, is defined in terms of the relationship of an individual with other people in a positive way [72]. Based on this concept, one only becomes a person through other people, as expressed in its “Nguni Expression” [73]. The Ubuntu concept also highlights that the most supreme obligation of mankind is to others. One needs to act in a benevolent, just, and truthful way to realize one’s humanity. Grange [72] also noted that community is the other dimension of the Ubuntu concept, where harmonious living following community values is emphasized as deeper relationships with other people. Coghlan and Brydon-Miller [73] said that Ubuntu can best be seen as a social philosophy with its grounding in taking care of family and the community, living in harmony with other people, and being hospitable to other people, being respectful, and expressing a sense of community. Ubuntu also underlines the need for collaboration and cooperation to build a strong community. Altruism is advanced through Ubuntu, harmony, and synergy between humanity and nature. The moral obligation that is domiciled in Ubuntu extends not only to other humans but also to other non-human entities such as the environment. Coghlan and Brydon-Miller [73] said that the Ubuntu concept is an alternative to individualistic and utilitarian concepts, which have their roots in Western countries. Ubuntu is being advanced in an African Renaissance, especially in different reformations that are taking place in education and public service. Van Breda [74] stated that the Ubuntu concept advocates for the development of ethics, sustainability, and an eco-spiritual attitude.

Closely linked to Ubuntu is Ukamu, which, according to Murove [75], is a concept that encompasses relatedness to the larger universe. This concept advances the idea that there is an ecological togetherness that is forged by the relationship between humans, ecology, and the spiritual being. Tangwa [76] confirmed this assertion when he said that Africa is convinced that all of the cosmos is an intricate interrelation of humans, plants, earth, and animals. Alluding to the Ubuntu and Ukamu concepts, Grange [72] stated that the goal of education should be to create personhood among learners with a focus on the community in the natural world so that they can value their shared destiny in society and the elements that safeguard the continuity of their community.

Ubuntu and Ukamu offer alternative worldviews for social transformation toward sustainability, downplaying economic incentives. Markets are crowding morals, and competition is relegating environmental issues to secondary positions, giving way to the growth of vices. Ubuntu and Ukamu ideologies create fully rational beings whose priorities are anchored in virtues. Through the advocacies that Ubuntu and Ukamu represent lies the potential of developing a moral consciousness in people that will drive a positive attitude towards social relations and the environment.

### 5.3. Moral Consciousness Development through Environmental Education

Environmental education contributes to the development of moral consciousness. According to Jickling and Spork [35], the actions being adapted to save nature in the world reflect societal ethics, with the accords and treaties being made on environmentalism amplifying the clarion call for a more ethical humanity. Felber [54] advocated for the creation of an economy that is inclusive through democratic systems of governance, sustainability, social justice, and dignity for all people, implying that morality and sustainability work in collaboration. Zsóka and Ásványi [77] found that education on sustainability develops instructions into value-based conduct and actions. The social support in which an individual exists is responsible for shaping an individual’s personality [61]. Kowasch and Lippe [19] said that when learners are engaged in an inquiry into consumption and production, values and morality become part of the dialogue.

Environmental education is a recipe for advancing ideologies that line up with virtue. Forgas and Jolliffe [29] researched the relationship between environmental concerns with political attitudes and libertarian attitudes. The research found that greater environmental concern had a direct correlation with radical political views. The radical political views identified include a lack of ethnocentrism, anti-free enterprise rhetoric, and economic conservatism. Further, the research also found that higher environmental concerns were associated with libertarian attitudes, which cut across different ethical and moral spheres. Such libertarian attitudes include advocacy for more ethical governance and equality. Forgas and Jolliffe [29] traced the genesis and the progression of environmentalism over the last century, and according to them, environmentalism gained most prominence in the 1960s. Furthermore, the efforts to care for the earth are congruent with traditional ideals, and to some extent, they are a component of religious moral practice. There is an association between the rampant environmentalism that emanated and the social libertarianism that occurred in the 1960s, especially in the West [78].

## 6. Recommendations for Environmental Education

According to Littledyke [10], cognitive development is most eminent in early childhood development and adolescent years. Through this stage, ideas are assimilated into the brain’s schemas in a way to adapt to the existing environment. As brains undergo biological maturation, the cognitive conflicts emanating from conflicting environmental information become ironed out, and individuals become aware of their learning. Through this tenet, it becomes apparent that moral reasoning and environmental education impressed upon the developmental brain go through maturation over the developmental stage and consequently become part of the mental framework in later life. It is imperative that during this developmental stage, great effort should be applied towards environmental awareness, forging environmental literacy, fostering environmental responsibility, and growing environmental competence [48]. Pedagogical initiatives that have environmental concerns should be introduced in this developmental stage to influence the children’s moral and cognitive development [10].

Individual behavior and reasoning are influenced by several factors, which include age, culture, intrinsic motivations, environment [29], social pressure, and information that is available to them [64,65,66,79]. As such, environmental education needs to be holistic so that environmental consciousness can be manifested in all the different facets of an individual. Reducing the monotony and rigidity that is associated with lectures and lessons on environmental responsibility is one of the ways of making it holistic [52]. The internalization of environmental education is forged by personal experience and by presenting it in an interdisciplinary manner. Using environmental challenges that challenge the ability of individuals to respond responsibly positions the environment as being worthy of important social action [28]. Actions such as watering plants and protecting trees from deforestation are tangible accomplishments that help to build a positive attitude and commitment toward environmental sustainability. The labor that the students put into environmental conservation will also teach them respect for labor and strengthen their belief that physically working to improve the environment is a worthwhile cause. “Reduce, Re-use, and Re-cycle” strategies should be encouraged among learners. Further, the learners will learn to shift the focus of their actions from themselves and to expand it to the environment, which is an element of selflessness [52].

Environmental education campaigns should be launched in schools, where the students can be equipped with environment-protection knowledge and participate in taking care of plants and the environment [52]. Participation in environment-protection day and consequent activities will strengthen the collective spirit and mental attitude toward environmental sustainability. Another educative process is organizing periodic symposiums and conferences on sustainability. These could also create excitement about conservation among learners as they exchange ideas about sustainability [80]. Organizing green exhibitions for students is also a proactive way of approaching environmental education. Learners can present projects and give speeches on their environmental ideas. According to Jie [13], learners educate themselves through organizing and performances. Another potent idea is the organization of summer camps that are structured around environmental protection. Fun activities that involve nature, conservation of resources, and keeping nature clean would help to prop up the personal and collective responsibility towards the environment. Participation in non-governmental organizations among learners should be encouraged, as they get to participate in environmental sustainability on a larger scale [81]. The above proactive efforts must be cyclic to keep environmental issues and commitment toward sustainability alive in the students’ minds until it becomes an integral part of their thought processes.

Active environmental learning and education should be scaled based on the level of understanding and commitment of the learners. At the infancy stage of environmental learning and for lower grade school, learners could be requested to take care of a flower bed, a lawn, or a few trees. Leaners in higher grade schools can be assigned larger environmental areas for which to care. Friendly competitions among the students and classes on who took care of their assigned sections would also help develop environmental responsibility and collective responsibility. To further strengthen environmental learning, environmental programs can be expanded to the community, where the learners take care and participate in community environmental programs and activities. The learners can be assigned pieces of the community’s green pastures to care for through cutting grass, watering and trimming trees, or even picking leaves [81].

The environment within which students exist should also inspire environmental sustainability. This can be done through the implementation of green campuses and spaces in learning institutions. Green technology in campuses and other institutions of learning should be used, and this includes the use of solar energy, and recycling [81]. There should be open spaces that have trees, waterways, lawns, and flower beds, creating a micro-climate within the campus premises and its surrounding. The learners should navigate through these ecosystems as they go about their studies and other activities, creating a feeling of oneness with nature. Such ecosystems will also enrich the quality of life of the learners on the campus [82]. De Graaff and Kolmos [83] highlighted that effective learning should adopt a problem–solution framework and also a project-based framework.

Problem-solving techniques should be integrated as part of the learning process to inspire the learners to become curious and problem solvers. Further, the problem-solving learning process should focus on real-life case scenarios and practical aspects that can be implemented within the learning environment and surrounding community [83]. Lindberg et al. [84] advocated for the use of the design thinking approach, which is a human-centered approach to designing educational programs. This approach is a conglomeration of different disciples that works iteratively to spur a culture of innovation. At the center of the design is a focus on the individuals that are involved. Specifically, an examination of the focus groups, their driving force, and aspirations is undertaken, after which the programs are created and are tailor-made on the focus groups. The design thinking approach is a problem-solution approach and seeks to drive frameworks that serve a specific group of individuals or societies [37].

Castro and Jabbour [8] presented a framework for a sustainable campus that can also be used to evaluate the level of sustainability in campuses that are used globally. The framework has three factions strands: first is university EMS (environmental management services), second is public participation and social responsibility, and the third is sustainable teaching and research. The EMS is a constituent of the environmental management and improvement processes that reduces the carbon footprint of the campus and fosters a green campus. The public participation and social responsibility element involve the formation of a partnership among all the stakeholders of the university to advance public participation, community service, and social justice. Sustainability teachings involve educational activities that include coursework and curriculum, research and development, conferences, seminars, and workshops [8].

## 7. Implications and Conclusions

The combination of moral and environmental education eliminates one-sided indoctrination, creating a cosmopolitan set of ideologies that offers justification for avoiding harm and ensuring justice, rights, and welfare of the environment. The emanation of environmentalism as a desideratum to drive the conscience in knowledge and pursuit at an individual as well as a communal level is the sole purpose of environmental education. On the other hand, moral education seeks to change the personality of individuals so that they can be aligned with the values, attitudes, and behaviors that drive environmentalism. Contrary research indicates that environmental knowledge cannot change the behavior of people towards the environment in isolation. However, when environmental knowledge is coupled with moralistic values, there is a possibility of change in individual behavior towards the environment.

This research has found that environmental education is largely deficient at the university level and at high school and grade school levels as well. Most learners have pedestrian knowledge and a low level of commitment to environmental issues. The partial understanding and efforts that most learners exhibit are inherent to the accrued experiences about environmentalism during their studies. The learning period is a formative stage for learners, where their personalities, personal values, and behavior are forged, which directs them for most of their later life. Moral and environmental education must be instilled in them in these formative years of their lives to create a conscience that will always foster environmental responsibility. Significantly proactive environmental education is more potent as an educational instrument as compared to passive environmental education. The integration of concepts such as Ubuntu and Ukamu in education systems will facilitate an intrinsic change in personality and perceptions towards environmentalism. Further, environmental education should also be extended to the community to facilitate the integration of ethics, values, skills, and attitude toward sustainability.

## Data Availability

The data presented in this study are available on request from the corresponding author. The data are not publicly available due to privacy.
